# Activated hepatic stellate cell-derived small extracellular vesicles facilitate M2 macrophage polarization and hepatoma progression via miR-27a-3p

**DOI:** 10.3389/fimmu.2024.1489679

**Published:** 2024-12-17

**Authors:** Yufeng Sun, Xiaoqian He, Jiayi Han, Wenxuan Yin, Haichen Wang, Jing Li, Weiqi Liu, Xingwang Kuai, Jiaying Lv, Juling Ji

**Affiliations:** ^1^ Department of Pathology, Medical School of Nantong University, Nantong, China; ^2^ Key Laboratory of Microenvironment and Translational Cancer Research, Nantong, China

**Keywords:** hepatic stellate cell, hepatoma, miRNA-27a-3p, tumor-associated macrophages, extracellular vesicles

## Abstract

The progression of hepatoma is heavily influenced by the microenvironment. Tumor-associated macrophages (TAMs) are considered to play a critical role in the tumor microenvironment (TME) and increase the aggressiveness of hepatoma. The activation of hepatic stellate cells (HSCs) is involved in hepatoma progression, and accumulating evidence demonstrates a change in microRNA (miRNA) expression during HSC activation. Therefore, the potential roles of HSCs-related miRNAs in macrophage differentiation and hepatoma progression deserve to be explored. The present study aimed to investigate the effects of miRNAs carried by small extracellular vesicles (sEVs) released by activated HSCs on hepatoma progression. The results indicated that miR-27a-3p was significantly upregulated in cells and corresponding sEVs during the activation of primary rat HSCs and human HSC line-LX2 cells. Furthermore, miR-27a-3p contributed to the proliferation and migration of hepatoma cells and promoted M2 polarization of macrophage. HSC-sEVs overexpressing miR-27a-3p can directly facilitate tumor progression and modulate macrophage polarization, indirectly contributing to hepatoma progression. Finally, Sprouty2 (SPRY2) was verified to be the target gene of miR-27a-3p. In conclusion, activated HSC-derived sEVs with high levels of miR-27a-3p might induce M2 macrophage polarization and promote hepatoma progression, providing new insights into the mechanism of hepatoma progression.

## 1 Introduction

Primary liver cancer is the second leading cause of cancer-related death worldwide, with approximately 841,000 new cases and 782,000 deaths each year (1). Hepatoma is composed of a complex collection of cancer cells and nonparenchymal cells, including tumor-associated macrophages (TAMs), cancer-associated fibroblasts (CAFs), and regulatory T cells, which contribute to a supportive tumor microenvironment (TME) (2, 3). TAMs infiltrate the tumor microenvironment and may differentiate into M2 immunosuppressive type, thereby promoting the development of hepatoma (4).

About 80% of hepatoma occurs on the basis of cirrhotic liver (1). Hepatic stellate cells (HSCs), which are liver-specific mesenchymal cells residing within the perisinusoidal space of Disse, play a crucial role in hepatic fibrosis. In a healthy liver, HSCs are quiescent and constitute the body’s largest reservoir of vitamin A. However, during liver injury, quiescent HSCs were activated and transformed into myofibroblasts that secrete matrix proteins, thereby leading to the development of liver cirrhosis (5). A recent study also confirmed HSCs as the major source of cancer-associated fibroblasts in hepatocellular carcinoma (HCC) (6). Considering the close relationship among liver fibrosis, cirrhosis, and hepatoma, activated HSCs may accelerate the progression of hepatoma through stroma-tumor interactions (7).

By analyzing our previous proteomic study of primary rat HSCs found that the most enriched biological function of the 200 proteins down-regulated during HSCs activation was involved in the immune response (8). We proposed that activated HSCs might affect the progression of hepatoma by participating in the immunosuppressive microenvironment. In a study later, we confirmed this hypothesis in liver cancer and para-cancer liver tissue samples from patients. Activated HSCs in para-cancer liver tissue promoted the progression of liver cancer by inducing an M2 immunosuppressive phenotype in macrophages (9, 10). However, the way in which HSCs affect the surrounding environment and the cells therein is still unclear.

Small extracellular vesicles (sEVs) are a group of nanovesicles mainly derived from endosomes with a diameter ranging from 30 to 150 nm, also known as exosomes (11, 12). They are secreted by the majority of cell types, loaded with DNAs, RNAs (including miRNA), proteins, and even metabolites from parent cells, constituting an essential intercellular communication (11, 12). Tumor cell-derived sEVs can induce the development of a tumor-promoting microenvironment by acting on surrounding stromal cells and facilitating signal transduction between cells (8, 13). There is an escalating interest in the role of stromal cell-derived sEVs in tumor progression.

MicroRNAs (miRNAs) are essential cargoes for sEVs (11, 12). They are a class of small noncoding RNAs that regulate gene expression by inhibiting the mRNA translation or the stability of targeting mRNA. They are involved in various biological and pathological processes (14). We previously reported that miR-27a-3p was upregulated in activated HSCs and promoted HSCs proliferation (15). It is worth further investigation if miR-27a-3p in sEVs derived from activated HSCs increases simultaneously, whether HSC-sEVs with higher expression of miR-27a-3p can affect the progression of hepatoma, and whether macrophages are involved.

In this study, we investigated the role of miR-27a-3p carried by activated HSC-derived sEVs in hepatoma progression, elucidating their potential and underlying mechanism in regulating tumor cells and the liver microenvironment.

## 2 Materials and methods

### 2.1 Biological materials

The human hepatic stellate cell line LX2 was provided by Feldman Laboratories. The human mononuclear cell line THP1 and human hepatoma cell lines (HepG2 and Huh7) were purchased from the Cell Bank/Stem Cell Bank of the Committee for Typical Cultures Preservation of Chinese Academy of Sciences. LX2 cells, HepG2 cells, and Huh7 cells were cultured in DMEM with 10% FBS (Gibco, USA). THP1 cells were cultured in 1640 (Invitrogen, USA) culture medium supplemented with 0.05 mM β-mercaptoethanol (Invitrogen, USA) and 10% FBS. Penicillin streptomycin (1%) was added to the above cell culture medium. Conventional culture was performed in an incubator at 37°C and 5% CO_2_.

Male Sprague-Dawley rats (body weight 450-550 g) and 5-week-old male BALB/c nude mice were housed in the animal facility of Nantong University. All animal experimental protocols were approved by the Animal Ethics Committee of Nantong University. Animal care and experiments were performed in line with the principles of the Guide for the Care and Use of Laboratory Animals formulated by Nantong University.

### 2.2 Primary HSC isolation, purification, and identification

The nonparenchymal rat liver cell suspension was obtained using a two-step enzymatic digestion method. HSC-enriched cells were obtained from these cells by density gradient centrifugation as described previously (8). HSCs were seeded in 25 cm^2^ culture flasks and cultured in DMEM (Gibco, USA) supplied with 10% exosome-depleted FBS (System Biosciences, Inc., USA) at 37°C in a 5% CO_2_ atmosphere incubator. The culture medium was replenished every 3 days. The culture media from Day 3, Day 11, and Day 14 were collected for the isolation of HSC-derived sEVs. HSCs were collected and lysed in RIPA buffer (Beyotime Biotechnology, China) or TRIzol (Life Technologies, USA) for protein or RNA sample preparation on Day 3 and Day 11.

Primary HSCs grown on coverslips were incubated with monoclonal antibodies against α-SMA (Abcam, UK), which is a key marker for HSCs activation. Then, the cells were incubated with Cy3-labeled secondary antibodies (Abcam, UK) at room temperature for 45 min, and nuclei were counterstained with Hoechst 33258 (Sigma-Aldrich, USA). Images were acquired using a fluorescence microscope (Olympus, Japan) (**Supplementary Figure S1**).

### 2.3 LX2 activation model

Human HSCs cell line LX2 was used in the present study (16). Cells (5×10^5^) were seeded in a 25 cm^2^ culture flask. After 24 h, 2% FBS DMEM culture medium was replaced, and 10 ng/mL of Recombinant Human TGF-β1 (Peprotech, USA) was added (17, 18). LX2 activation was induced, and the same volume of PBS buffer containing 5% saline solution (TGF-β1 buffer) was added as the negative control. After 24 h, the cells and supernatant were collected and verified by qRT-PCR, and western blotting was performed 48 h later.

### 2.4 Western blotting

Protein samples were separated using 10% sodium dodecyl sulfate-polyacrylamide gel electrophoresis and subsequently transferred onto PVDF membranes (Millipore). After blocking with 5% skim milk at room temperature for 1 h, the membranes were incubated with primary antibodies at 4°C overnight, and then incubated with the appropriate horseradish peroxidase-conjugated secondary antibody for 1 h at room temperature. Total protein staining with β-actin served as the loading control. The protein bands were treated with enhanced chemiluminescent (ECL) substrate (Tanon, China) and imaged using a chemiluminescence imaging system.

### 2.5 Cell transfection

Lipofectamine 2000 Transfection Reagent (Invitrogen, USA) was used to transfect cells. Hsa-miR-27a-3p mimic (mimic-miR-27a-3p), has-miR-27a-3p inhibitor (anti-miR-27a-3p), negative controls mimic NC #22 (mimic-NC), and inhibitor NC #22 (anti-NC) were from RiboBio (RiboBio Biotechnology, China). In the present study, according to the manufacturer’s recommended dosage and our preliminary experiment, a final concentration of 75 nM was used for all the chemically modified small molecular nucleic acids.

### 2.6 Nude mouse xenograft model

A total of twenty 5-week-old BALB/c male nude mice weighing 20g were divided into four groups, and each group contained 5 mice. HepG2 cells transfected with mimic-miR-27a-3p, mimic-NC, anti-miR-27a-3p, or anti-NC were subcutaneously inoculated into the forelimbs of nude mice. Each mouse was injected with 5×10^6^ cells. The size and weight of tumors were measured at 7, 14, 21, and 28 d. After 28 days, mice were euthanized, and tumors were collected for analysis (evaluation of tumor volume and weight). Immunohistochemical staining was performed on paraffin sections of tumor samples with Envision+ kits (Dako, USA) according to the manufacturer’s instructions. Monoclonal antibodies against CD206 (Boster, China), CD68 (Boster, China), Ki67 (Abcam, USA), and SPRY2 (Proteintech, China) were used. Images were acquired with a fluorescence microscope. The schematic diagram of the study design is shown in **Supplementary Figure S2**.

### 2.7 Purification of sEVs from the conditioned medium

The supernatant was collected and centrifuged at 3000 g at 4°C for 15 min, and then the precipitate (cells and cell debris) was removed. The supernatant was filtered through a 0.22 μm filter and centrifuged at 4000 g for 30 min at 4°C using an Ultra-15 centrifugal filter (Merck, Germany). The exosome-enriched sEV fractions were obtained from the concentrated mediums by ultra-centrifugation at 100,000 ×g for 90 min (Hitachi Ltd., Japan), then the pellet was washed with PBS, followed by a second step of ultracentrifugation at 100,000 ×g for 90 min (all steps are performed at 4°C). Afterward, the supernatant was discarded.

To increase the recovery rate, the concentrated mediums were mixed well with Tissue Culture Media SEVs Precipitation Solution (System Biosciences, Inc., USA) and incubated overnight in the dark at 4°C (19). Then, the supernatant was centrifuged at 1500 g at 4°C for 30 min, and the supernatant was discarded. The supernatant was aspirated, and the sEV precipitate was collected.

After adding 100 μl granule-free PBS to resuspended sEVs in the precipitate, nanosight NS300 (Malvern, UK) was used to measure the particle size and number of sEVs (20). The obtained sEVs were identified by transmission electron microscopy (TEM) (19). The schematic diagram of study design is shown in **Supplementary Figure S3**.

### 2.8 Quantitative reverse transcription polymerase chain reaction

Total cellular RNA and sEVs RNA were extracted by TRIzol Reagent and TRIzol LS Reagent (Ambion, USA), respectively. The spiking-in-cel-miR-39 was added to Trizol at the step of sEVs lysis; the final concentration of cel-miR-39 is 10 fmol. Reverse transcription of the mRNA was performed according to the PrimeScript 1st Strand cDNA Synthesis Kit (TaKaRa, Japan) reagent instructions. According to the reagent instructions of the miRNA RT–PCR Starter Kit (RiboBio, China), reverse transcription of miRNA was performed to synthesize cDNA. To quantitatively test mRNA and mature miRNAs, cDNA templates and primer sets were mixed with TB Green Premix Ex Taq ii (Takara, Japan) and corrected with ROX dye. The expression levels of mRNA and miRNAs in cells were normalized to those of GAPDH and U6 snRNA, respectively, and exosomal miRNA levels were normalized to cel-miR-39 levels.

### 2.9 Transwell migration assay

The migration ability was assessed through a transwell chamber assay (Millipore, USA). The transfected cells were serum-starved for 4 h, then seeded into the upper chamber with serum-free medium (8×10^4^ cells per well); the bottom of the chamber contained the mixed culture solution with 5% FBS according to experimental groups; these plates were then cultured in a 37°C, 5% CO_2_ incubator for 24 h. The supernatant was discarded and washed three times with 1× PBS. Then, the cells were fixed with 2% paraformaldehyde at room temperature for 10 min, stained with 0.1% crystal violet solution, and incubated at room temperature for 10 min. The cells were washed with ultrapure water 3 to 5 times after each step, and the cells in the upper chamber were removed with a medical-grade cotton swab. The number of migrated cells was counted using a microscope.

### 2.10 Luciferase assay

After 24 hours of seeding into 24-well plates for transfection, cells were transiently transfected with 2 μg psiCHECK-2/Sprouty2 (SPRY2) x3 reporter plasmid and 75 nM mimic-miR-27a-3p or mimic NC by Lipofectamine 2000 (Invitrogen, USA). A dual-luciferase reporter system (Promega, USA) was used to perform luciferase assays 42 h later. A Veritas Microplate Luminometer (Turner Biosystems, Sunnyvale, USA) was used to detect Renilla and firefly luciferase signals.

### 2.11 Transfection of SPRY2 siRNA and SPRY2 overexpression plasmid

The siRNA fragments targeting SPRY2 and negative control siRNA (Genepharma, China) were transfected into HepG2 or Huh7 cells using Lipofectamine 2000 (Invitrogen, USA) at a final concentration of 50 nM. Twenty-four hours later, these cells were subsequentially transfected with mimic-miR-27a-3p. The expression of SPRY2 was determined by western blotting. The cell function was evaluated using the CCK8 assay and transwell assay.

The SPRY2 overexpression plasmids (pLV2-SPRY2 (human)-3×FLAG-Puro, OE-SPRY2) and negative control (pLV2-MCS-3×FLAG-IRES-Puro, OE-NC) were obtained from MiaoLing Biology (MiaoLingBio, China). Plasmids were transfected into HepG2 or Huh7 cells using Lipofectamine 2000 (Invitrogen, USA) at a final concentration of 2 μg per well. Twenty-four hours later, these cells were subsequentially transfected with anti-miR-27a-3p. The expression of SPRY2 was determined by western blotting. The cell function was evaluated using the CCK8 assay and transwell assay.

### 2.12 Fluorescence *in situ* hybridization

The paraffin sections of nude mice xenograft tumors were subjected to FISH assay using an FAM-labeled miR-27a-3p probe (Genepharma, China) according to the manufacturer’s instructions. Images were obtained by fluorescence microscope.

### 2.13 Statistical analysis

Quantitative data are expressed as means ± standard errors of the means (SEM). Student’s t-test or one-way ANOVA (GraphPad Prism 5.0) was used to perform statistical analyses unless otherwise stated. All P values were two-sided, and statistical significance was accepted at P < 0.05. Unless otherwise stated, all experiments were performed three times.

## 3 Results

### 3.1 miR-27a-3p expression was significantly upregulated in cells and sEVs derived from activated HSCs vs. quiescent HSCs

To compare miRNA expression in activated and quiescent HSC-derived sEVs, we established a widely recognized *in vitro* culture activation model of primary rat HSCs (21). In accordance with the phenotype, HSCs cultured for 3 days were designated as quiescent HSCs, while those cultured for more than 10 days (11d HSCs and 14d HSCs used in the present study) were regarded as activated HSCs (8) (**Supplementary Figure S1**). In previous studies, we also found that the expression of miR-27a-3p in primary rat HSCs was upregulated during activation (15). Based on the hypothesis that HSCs could act on adjacent environments through sEV secretion, we hypothesized that miR-27a-3p would also be upregulated in sEVs secreted from activated HSCs.

**Figure 1:**
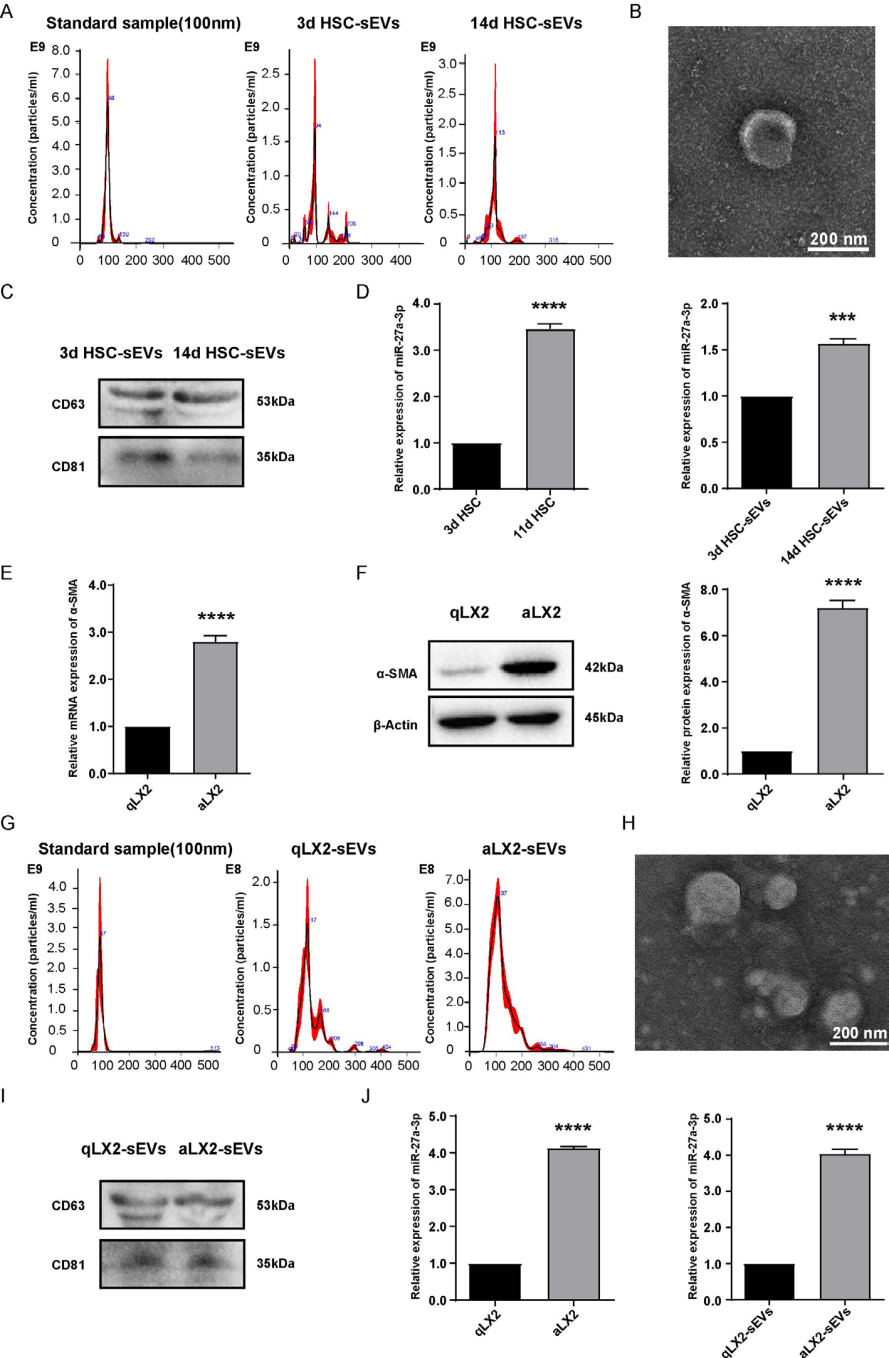
miR-27a-3p expression was increased in cells and sEVs of activated HSCs and aLX2 cells vs. their quiescent counterparts. **(A–C)** NTA, TEM, and western blotting analysis of sEVs isolated from 3 day quiescent HSCs and 14 day activated HSCs. NTA instrument (NanoSight NS300) was calibrated with 100 nm standard. The expression of the characteristic sEVs molecules CD63 and CD81 was detected by western blotting. **(D)** qRT-PCR was used to detect the expression of miR-27a-3p in cells and sEVs from quiescent (day 3) activated primary rats HSCs (day 11 and day 14). U6 snRNA served as internal reference for cell samples and cel-miR-39 served as external reference for sEVs samples (mean ± SEM). ***P < 0.001, ****P < 0.0001 vs. Control; **(E, F)** The expression of α-SMA in LX2 cells treated with 10 ng/ml TGFβ1 for 24 h for mRNA as detected by qRT-PCR normalized to GAPDH; or 48 h for protein as detected by western blotting normalized to β-actin (mean ± SEM). ****P < 0.0001 vs. Control; **(G–I)** NTA, TEM, and western blotting analysis of sEVs isolated from activated LX2 cells. NTA instrument was calibrated with 100 nm standard. The expression of the characteristic sEVs molecules CD63 and CD81 was detected by western blotting; **(J)** qRT-PCR was used to detect the expression of miR-27a-3p in qLX2 and aLX2 cells and supernatant sEVs in LX2-activation models. U6 snRNA served as internal reference for cell samples and cel-miR-39 served as external reference for sEVs samples (mean ± SEM). ****P < 0.0001 vs. control. 3d, 3 days; 11d, 11 days; 14d, 14 days; qLX2, quiescent LX2; aLX2, activated LX2. Data were from three independent tests.

To prove this hypothesis, we prepared RNA samples from 3-day quiescent HSCs (3d HSCs) and 14-day activated HSCs (14d HSCs) and sEVs from the corresponding culture medium. HSC-derived sEVs were identified by nanoparticle tracking analyses (NTA) to show the particle size distribution and the average diameter of the particles ranged from 90 nm to 120 nm (**Figure 1A**). The shape of sEVs was spherical or cup-shaped as identified by transmission electron microscopy (TEM) (**Figure 1B**). To further confirm that the isolated particles were indeed exosome-enriched sEVs, the expression of generally recommended exosome biomarkers was examined by western blotting (**Figure 1C**). The expression levels of miR-27a-3p in the cellular and supernatant sEVs of primary rat HSCs were compared by qRT-PCR. We found that miR-27a-3p was not only upregulated in activated HSCs but was also upregulated in the sEVs secreted from cells (**Figure 1D**).

To verify that the upregulation of miR-27a-3p in activated HSCs is a cross-species event, the human hepatic stellate cell line LX2 was selected to establish an *in vitro* activation model of HSCs. According to a previous study, α-smooth muscle actin (α-SMA), a marker for the activation of HSCs, was upregulated during the activation of HSCs (22). LX2 cells were cultured with 2% FBS DMEM culture solution supplemented with 10 ng/mL TGF-β1 cytokine, which is a robust driver of HSC activation for 24 h and 48 h (the same volume of buffer for TGF-β1 was added to the negative control group). The results showed that the expression of α-SMA was significantly higher in the activated LX2 group (TGFβ 1-treated activated group, aLX2) than in the quiescent LX2 group (negative control group, qLX2), which confirmed the success of the *in vitro* HSC activation model (**Figures 1E, F**). The sEVs derived from LX2 cells were isolated, purified, and then identified by TEM, western blotting, and NTA (**Figures 1G–I**), thereby confirming that the isolated particles were sEVs.

The expression of miR-27a-3p was detected by qRT-PCR, and the data showed that miR-27a-3p was upregulated in both aLX2 cells and sEVs secreted from them (**Figure 1J**). We verified the results in primary HSCs from rats in the activation model of human HSCs.

### 3.2 Overexpression of miR-27a-3p correlated with increased proliferation and migration capability of hepatoma cells

**Figure 2:**
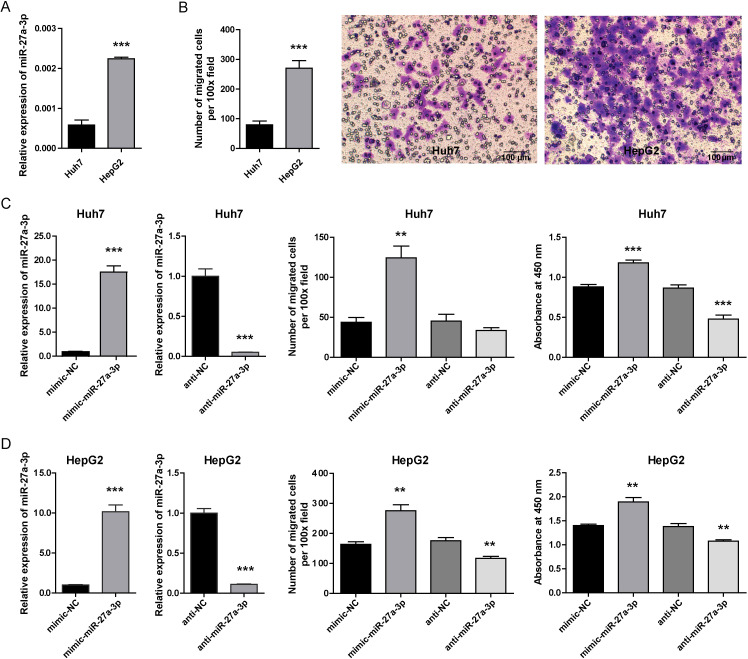
The expression of miR-27a-3p in hepatoma cells and the influence of miR-27a-3p on cellular behavior. **(A)** The expression of miR-27a-3p in the human hepatoma cell lines Huh7 and HepG2 was evaluated by qRT-PCR and normalized to that of U6 snRNA (mean ± SEM). ***P < 0.001 vs. control. **(B)** The migration ability of Huh7 and HepG2 cells. The number of migrated cells was counted manually after crystal violet staining (mean ± SEM), and representative images are provided, bar = 100 μm. ***P < 0.001 vs. control. **(C, D)** The expression of miR-27a-3p in hepatoma cells transfected with mimic-miR-27a-3p was significantly increased, and cells transfected with anti-miR-27a-3p were significantly down-regulated (left). The number of migrated cells was counted manually (middle). Cell proliferation capacity was evaluated by CCK-8 assay 36 h after mimic-miR-27a-3p and anti-miR-27a-3p transfection, and the absorbance at 450 nm is shown. (right) (mean ± SEM). **P < 0.01, ***P < 0.001 vs. control. The data were from three independent experiments.

To clarify the role of miR-27a-3p in hepatoma, the human liver cancer cell lines Huh7 (low miR-27a-3p expression) and HepG2 (high miR-27a-3p expression) were selected (**Figure 2A**). In our previous study, we found that the proliferation ability of HepG2 cells that highly expressed miR-27a-3p was higher than that of Huh7 cells (23). The migration ability of HepG2 cells was also significantly higher than that of Huh7 cells, as detected by transwell migration assay (**Figure 2B**). To study the effects of miR-27a-3p on hepatoma cell biological behaviors *in vitro*, we transfected Huh7 and HepG2 cells with mimic/anti-miR-27a-3p or controls (left of **Figures 2C, D**). As determined by transwell migration assays, migration of both Huh7 and HepG2 cells transfected with the mimic-miR-27a-3p was significantly enhanced (Middle of **Figures 2C, D**). Meanwhile, the migration of HepG2 cells was suppressed by the anti-miR-27a-3p, but the impact of anti-miR-27a-3p on the migration of Huh7 cells was insignificant. The proliferation of Huh7 and HepG2 cells was promoted by the mimic-miR-27a-3p but decreased by the anti-miR-27a-3p, as demonstrated by CCK-8 assays (right of **Figures 2C, D**).

### 3.3 miR-27a-3p promoted tumor growth in a nude mouse xenograft model

**Figure 3:**
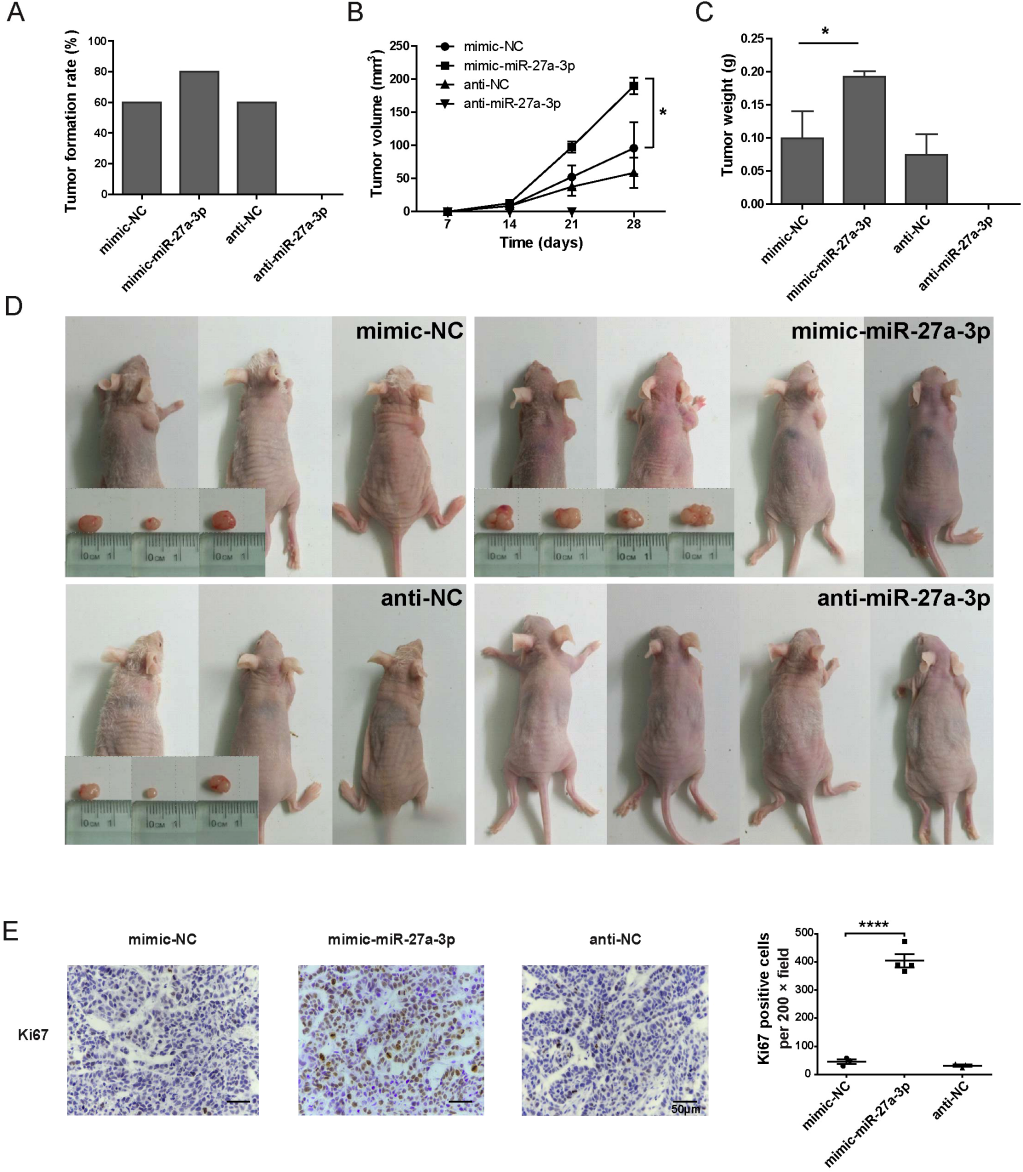
miR-27a-3p promotes hepatoma formation and growth *in vivo*. **(A)** Tumor formation rate by 2 weeks for each group of the nude mice xenograft model (n = 5). **(B)** The tumor volume by day 7, day 14, day 21, and day 28 in each group of nude mice xenograft model (mean ± SEM). Statistical analysis was conducted using Two-way Repeated-Measures ANOVA, *P < 0.05 vs. control. **(C)** The tumor weight of the nude mice xenograft model group at the time of sacrifice (mean ± SEM), *P < 0.05 vs. control. **(D)** Nude mice and xenograft tumors in each group, gross view, bar = 1 cm. **(E)** Representative images for the immunohistochemical staining of Ki67 for xenograft tumors, bar = 50 μm; and the number of Ki67 positive cells per 200 × field for each group (mean ± SEM). ****P < 0.0001 vs. control. The nude mice xenograft experiment was repeated once; the data presented were from one representative batch.

We established a nude mouse xenograft model. HepG2 cells transfected with mimic/anti-miR-27a-3p or controls were injected subcutaneously into male nude mice, which were divided into four groups (5 mice per group). After 2 weeks, 3 mice in the mimic-NC group and 3 mice in the anti-NC group formed xenograft tumors. Four xenograft tumors were formed in the mimic-miR-27a-3p group. No xenograft tumors were found in the 5 mice in the anti-miR-27a-3p group (**Figures 3A, D**). The tumor size was measured at 7 d, 14 d, 21 d, and 28 d. On the 28th day after injection, the tumor volume in the mimic-miR-27a-3p group was significantly larger than that in the mimic-NC group (P = 0.004) (**Figure 3B**). Correspondingly, the tumor weight of the mimic-miR-27a-3p group was approximately 195% of that of the control group. The difference in tumor volume and weight between the mimic-NC and anti-NC groups (P = 0.345) was not significant (**Figure 3C**).

To further verify the effect of miR-27a-3p on the proliferation of tumor cells, the expression of Ki67 was detected by immunohistochemistry. The Ki67-positive cells in all the 200-fold magnification fields of xenograft tumors from each group were counted. Due to the therapeutic effect of anti-miR-27a-3p, there was no tumor in the nude mice xenograft model after 28 days of anti-miR-27a-3p treatment. It turned out that the number of Ki67-positive cells was significantly increased in the mimic-miR-27a-3p group compared to the mimic-NC or anti-NC group (**Figure 3E**). These results suggest that miR-27a-3p promotes tumor proliferation to support a high degree of malignancy.

### 3.4 Overexpression of miR-27a-3p induced M2 polarization of macrophages

**Figure 4:**
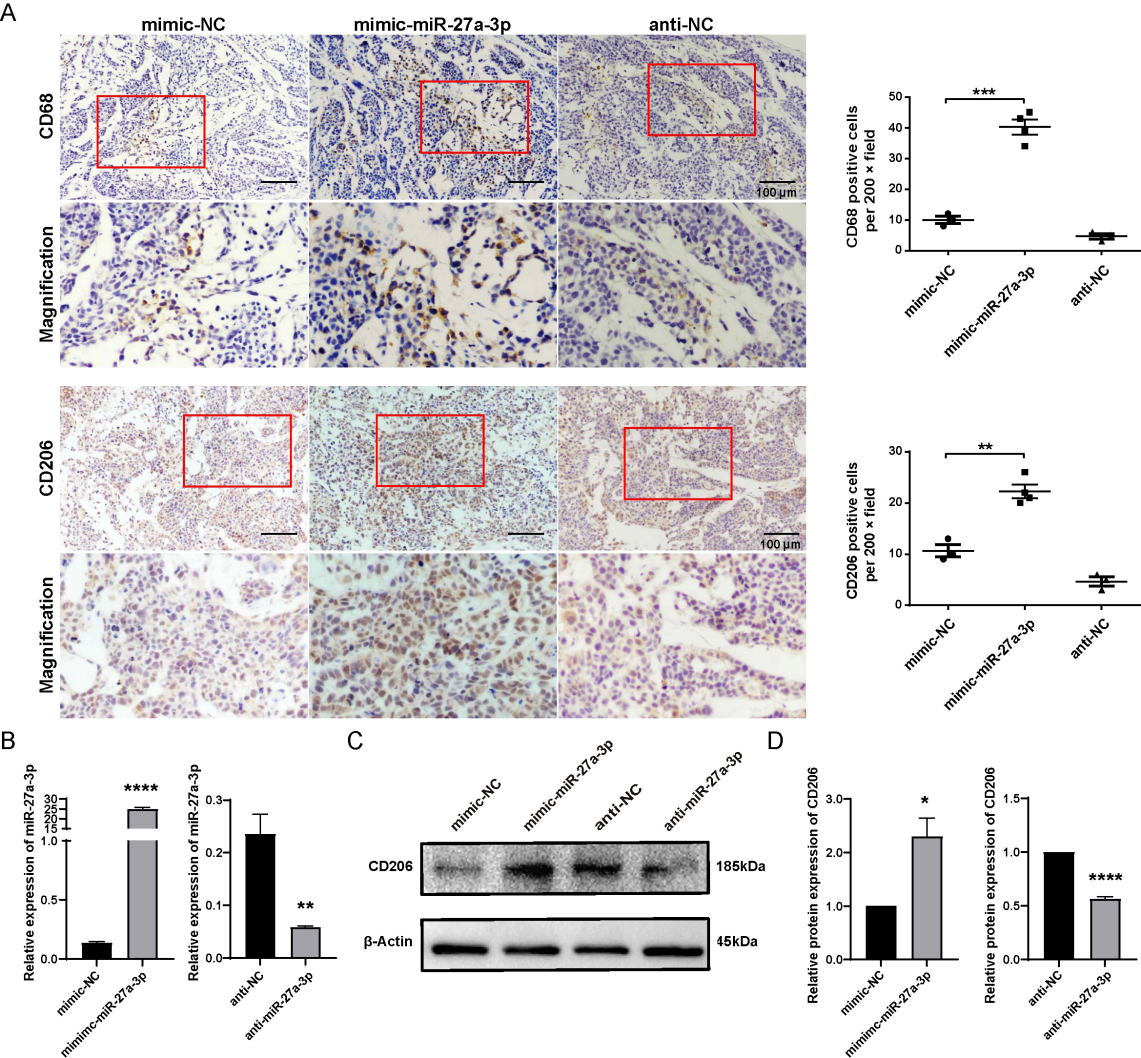
miR-27a-3p promotes the M2 polarization of macrophage. **(A)** Representative images for the immunohistochemical staining of macrophage maker CD68 and CD206 in nude mice xenografts, bar = 100 μm; and the number of CD68, CD206 positive cells per 200 × field for each group (mean ± SEM). **P < 0.01, ***P < 0.001 vs control. Below each group of images is an image showing the magnification of the corresponding box area above. **(B)** The expression of miR-27a-3p in mimic-miR-27a-3p or anti-miR-27a-3p treated THP1 cells was evaluated by qRT-PCR 24 h after transfection, normalized to U6 snRNA (mean ± SEM). **P < 0.01, ****P < 0.0001 vs. control. **(C, D)** The expression of CD206 was evaluated in mimic-miR-27a-3p or anti-miR-27a-3p treated THP1 cells by western blotting normalized to β-actin (mean ± SEM). *P < 0.05, ****P < 0.0001 vs. control. The data were from three independent experiments.

In the present study, CD68 was employed as the marker of tumor-infiltrating macrophages, while CD206 was utilized as the marker of M2-type macrophages. The result of immunohistochemistry indicated an elevated number of CD68 and CD206-positive cells in the mimic-miR-27a-3p group using the same batch of xenografts, suggesting that miR-27a-3p promotes the infiltration of liver macrophages and participates in the development of the TME. In addition, among the three groups, the mimic-miR-27a-3p group showed elevated CD206 expression, suggesting that M2-type macrophages in the tumor were dominant compared with M1-type macrophages (**Figure 4A**).

We further investigated whether overexpression of miR-27a-3p could polarize macrophages into the M2 type by using THP-1 cell lines. The phenotype of THP1-M0 cells transfected with mimic/anti-miR-27a-3p or controls was detected by western blotting. The transfection of mimic-miR-27a-3p effectively upregulated miR-27a-3p, and anti-miR-27a-3p effectively downregulated miR-27a-3p in THP1 cells (**Figure 4B**). Results showed that M2 marker (CD206) in cells transfected with mimic-miR-27a-3p were apparently upregulated (**Figures 4C, D**).

### 3.5 HSC sEVs highly expressed miR-27a-3p and promoted the migration of hepatoma cells

**Figure 5:**
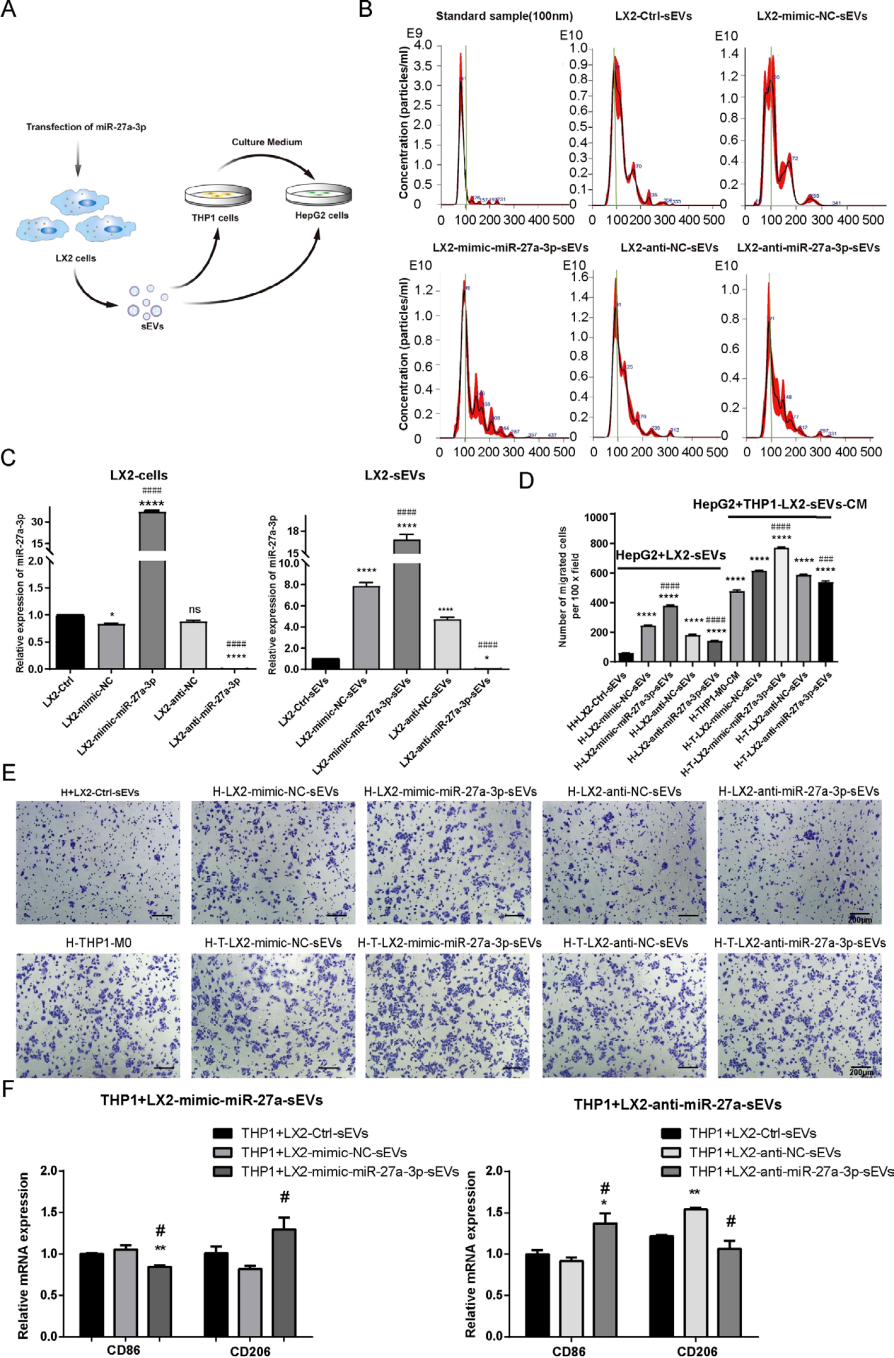
The miR-27a-3p over-expressing HSC-sEVs promote HepG2 cell migration directly or through educating macrophages. **(A)** Schematic of the conditioned culture experiments. **(B)** Representative images for the size distribution and concentration of sEV particles in the supernatant of transfected LX2 cells as detected by NTA. **(C)** The expression of miR-27a-3p in mimic-miR-27a-3p or an-ti-miR-27a-3p transfected LX2 cells and sEVs was detected by qRT-PCR, standardized to U6 snRNA for cell samples or cel-miR-39 for sEVs samples, respectively. *P < 0.05, ****P < 0.0001 vs. control. ####P < 0.0001 vs. NC. **(D, E)** Transwell migration assays for conditioned cultured. Representative images for the crystal violet staining of migrated cells, bar = 200 μm; and the number of migrated cells per 100 × field for each group (mean ± SEM). ****P < 0.0001 vs. control. ###P < 0.001, ####P < 0.0001 vs. NC. **(F)** The expression levels of surface markers in THP1 macrophages was detected using qRT-PCR. *P < 0.05, **P < 0.01 vs. control. #P < 0.05 vs. NC. H, HepG2 cells; qLX2, quiescent LX2 cells; aLX2, active LX2 cells; CM, culture medium. The data were from three independent experiments.

To further verify the role of miR-27a-3p carried by sEVs from activated HSCs in promoting the migration ability of hepatoma cells, we performed conditioned culture of HepG2 cells with miR-27a-3p over-expressing LX2-sEVs. Additionally, we also test the influence of miR-27a-3p over-expressing LX2-sEVs cultured macrophages on HepG2 cells (**Figure 5A**). LX2 cells were transfected according to groups (completely blank control group, mimic-NC group, mimic-miR-27a-3p group, anti-NC group, anti-miR-27a-3p group). After 24 h, we collected the supernatant from each group, and the sEVs were separated and purified. The particle size and particle number of sEVs were determined by NTA (**Figure 5B**), and the expression of miR-27a-3p in sEVs was assessed by qRT-PCR (**Figure 5C**).

Then, we added the sEVs highly expressed miR-27a-3p into the culture of HepG2 cells at a ratio of sEV particle number: cell number =10000: 1. Transwell assays showed that the sEVs of LX2 cells over-expressing miR-27a-3p facilitated the migration of HepG2 cells. To explore the influence of miR-27a-3p over-expressing LX2-sEVs treated macrophageson HepG2 cells, the THP1 cells were pretreated with 5 ng/ml PMA for 24 h to induce M0 macrophages. We added the miR-27a-3p over-expressing sEVs to the culture of THP1-M0 cells for 24 h, after which we collected the supernatant and added it to HepG2 cells culture. Transwell assays showed that the supernatant of THP1-M0 cells treated by miR-27a-3p over-expressing sEVs from LX2 cells further enhanced the migration of HepG2 cells (**Figures 5D, E**). In addition, the expression levels of surface markers in THP1 macrophages were detected using qRT-PCR, with CD86 and CD206 serving as specific markers for M1 and M2 macrophage subtypes, respectively (**Figure 5F**). The results showed that the expression of M1 phenotype markers (CD86) decreased after the treatment of sEVs derived from mimic-miR-27a-3p-treated LX2 while the expression of M2 phenotype markers (CD206) increased. Conversely, the opposite effect was observed after the treatment of sEVs derived from anti-miR-27a-3p-treated LX2. This indicates that sEVs released by LX2 cells overexpressing miR-27a-3p promote polarization of macrophages towards an M2 phenotype.

Based on these data, we concluded that aLX2 cell-derived sEVs containing miR-27a-3p establish a tumor-promoting microenvironment.

### 3.6 SPRY2 was a direct target of miR-27a-3p

**Figure 6:**
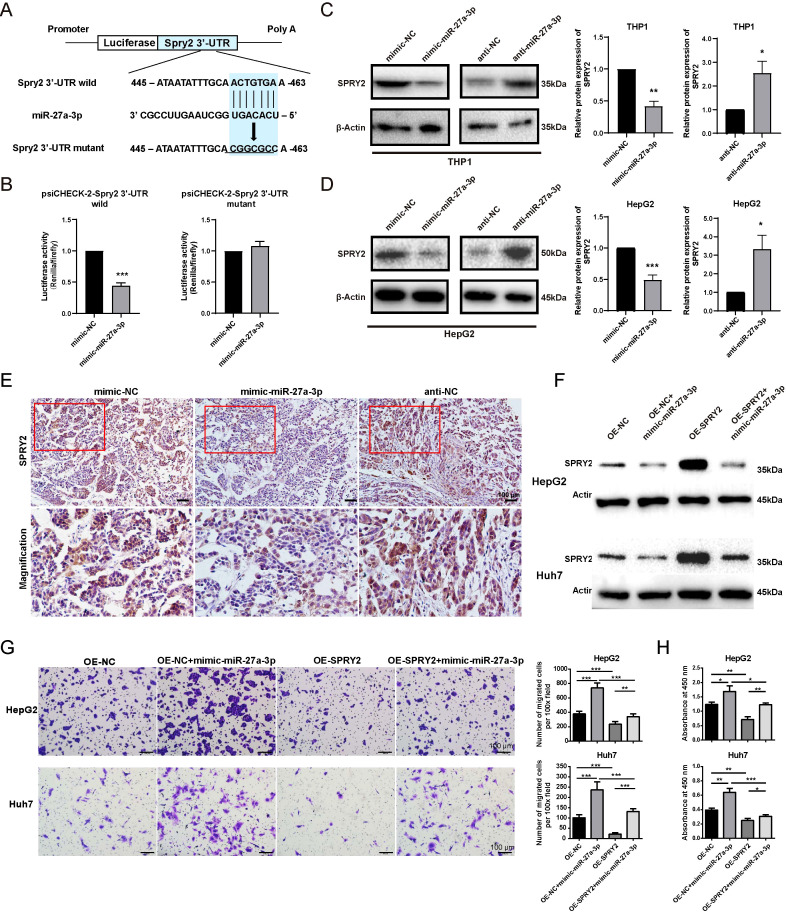
SPRY2 was a direct target of miR-27-a-3p in hepatoma. **(A)** Construct a map of psiCHECK-2/Spry2x3 used for the luciferase assay. The 3’-UTR of SPRY2 was cloned into the vector after the Renilla luciferase gene (hRluc). The firefly luciferase gene (hluc+) was expressed in the vector as an internal control. Seven nucleotides of the seed sequence were mutated, as shown in the figure, to construct the mutant vector. **(B)** miR-27a-3p directly acted on the 3’-UTR of SPRY2. HEK293T cells were cotransfected with mimic-miR-27a-3p together with a psiCHECK-2-Spry2 3’-UTR wild-type vector or psiCHECK-2-Spry2 3’-UTR mutant vector, and mimic-NC and vectors were transfected as controls (mean ± SEM). ***P < 0.001 vs. control. **(C, D)** The expression of SPRY2 in HepG2 and THP1 cells after transfection with mimic/anti-miR-27a-3p or NC was evaluated by western blotting. *P < 0.05, **P < 0.01, ***P < 0.001 vs. control. **(E)** Representative images for the immunohistochemical staining of SPRY2 in nude mice xenografts, bar = 100 μm. Below each group of images is an image showing the magnification of the corresponding box area above. **(F)** The expression of SPRY2 was evaluated by western blotting normalized to β-actin. HepG2 and Huh7 cells overexpressing SPRY2 were transfected with mimic-miR-27a-3p. **(G)** Transwell migration assay. HepG2 and Huh7 cells overexpressing SPRY2 were transfected with mimic-miR-27a-3p. **(H)** CCK8 proliferation assay. HepG2 and Huh7 cells overexpressing SPRY2 were transfected with mimic-miR-27a-3p. The absorbance at 450 nm is shown. (mean ± SEM). *P < 0.05, **P < 0.01, ***P < 0.001. The data were from three independent experiments.

To further elucidate the mechanisms by which miR-27a-3p is involved in the progression of hepatoma, we used an online miRNA target prediction tool TargetScan (https://www.targetscan.org), to screen the potential targets of miR-27a-3p. TargetScan predicts biological targets of miRNAs by searching for conserved sites that match the seed region of each miRNA. The miR-27a-3p belongs to a broadly conserved microRNA family. According to the website instructions, the following criteria were adopted for its target screen: i) Species, human; ii) Context++ score percentile ≥ 99; iii) probability of conserved targeting (PCT) ≥ 0.80. Among the predicted target genes, by the gene function annotation and literature reviewing, we further identified SPRY2, which is highly conserved among species for further verification (**Figure 6A**).

To confirm that SPRY2 is the target of miR-27a-3p, we separately cloned the full-length wild-type and mutant SPRY2 3’-UTRs into the psiCHECK-2 vector, after which the vectors with the mimic-miR-27a-3p or the control were transfected into HEK293T cells. We found that the luciferase activity of the psiCHECK-2 vector with the wild-type SPRY2 3’ UTR was inhibited by miR-27a-3p, but the psiCHECK-2 vector with the mutant SPRY2 3’-UTR was not affected. The results fully illustrated that SPRY2 can be directly affected by miR-27a-3p (**Figure 6B**).

Subsequently, the expression of SPRY2 in HepG2 cells and THP1 cells transfected with mimic/anti-miR-27a-3p was detected by western blotting at the protein level. The results revealed that SPRY2 expression in cells transfected with mimic-miR-27a-3p was dramatically reduced in comparison to that in cells transfected with mimic-NC but was increased in cells transfected with anti-miR-27a-3p (**Figures 6C, D**).

The expression of miR-27a-3p in hepatoma xenografts was detected by *in situ* hybridization technology. The results demonstrated that pretreatment with mimic-miR-27a-3p led to an upregulation of miR-27a-3p expression within hepatoma xenografts (**Supplementary Figure S4**). Subsequently, immunohistochemical staining was used to evaluate the expression level of SPRY2 in hepatoma xenografts. The results revealed that pretreatment with miR-27a-3p resulted in a downregulation of SPRY2 expression, suggesting a potential direct targeting relationship between miR-27a-3p and SPRY2 (**Figure 6E**).

HepG2 and Huh7 cells overexpressing SPRY2 were transfected with mimic-miR-27a-3p. The western blotting results demonstrated that the upregulation of SPRY2 in HepG2 and Huh7 cells, which were overexpressing SPRY2, was restrained by the transfection with mimic-miR-27a-3p (**Figure 6F**; **Supplementary Figure S5A**). In addition, the expression of SPRY2 in SPRY2-knockdown HepG2 and Huh7 was increased by anti-miR-27a-3p (**Supplementary Figure S5B**). The results of the transwell migration assay and CCK8 assay showed that the overexpression of SPRY2 in HepG2 and Huh7 partially alleviated the promoting effects of mimic-miR-27a-3p on cell migration and proliferation (**Figures 6G, H**). Similarly, transwell assay and CCK8 assay results indicated that the knockdown of SPRY2 in liver cancer cells relieved the inhibitory effects of anti-miR-27a-3p on cell proliferation and migration (**Supplementary Figures S5C, D**).

## 4 Discussion

The role of HSCs/CAF in the progression of liver cancer has received increasing attention in recent years. However, current research is primarily focused on the direct effect of HSCs on tumor cells. In quiescent HSCs, mitogen-activated protein kinase (MARK) is strongly phosphorylated, thus activating NF-kB and extracellular-regulated kinase (ERK) cascade signaling pathways (24), leading to an increased release of IL8, which promotes the invasion and metastasis of hepatoma (25, 26). Another study reported that HSCs can mediate hepatoma progression by releasing factors that promote epithelial-mesenchymal trans-formation and angiogenesis, such as VEGF, MMP2, MMP9, bFGF, and TGF-β (27, 28). In our previous study, we found that the activated HSCs might induce an immunosuppressive phenotype of macrophages, thus promoting the progression of hepatoma and leading to a poor prognosis (10). In the present study, focusing on the role of sEV in intercellular signaling, we further investigated the underlying mechanism.

The transportation of miRNAs mediated by sEVs is an important mechanism of genetic exchange among cells (12). The type and abundance of miRNAs loaded in sEVs changed synchronously with the functional state of the parent cells in most cases. We have reported that miR-27a-3p is upregulated in activated primary rat HSCs, which promotes the activation of primary rat HSCs by reducing fat accumulation and promoting cell proliferation (15). Several subsequent studies showed that the upregulation of miR-27a-3p can promote tumor proliferation and migration and accelerate the progression of different types of cancer, including breast carcinoma, renal cell carcinoma, cervical carcinoma, and hepatoma (17, 18, 23, 29).

According to the previous findings of our group and the relevant reports, the present study aimed to investigate the expression of miR-27a-3p in activated HSCs-derived sEVs, including culture-activated primary rat HSCs, and TGF-β1 activated human HSCs cell line-LX2. We also investigated the direct effects of miR-27a-3p over-expressing HSC-sEVs on hepatoma cell proliferation and migration and indirect effects on tumor cells through educating macrophages. We found that miR-27a-3p is upregulated in activated rat HSC-derived sEVs and TGF-β1 activated human LX2-derived sEVs. These findings confirmed that the upregulation of miR-27a-3p in activated HSCs and corresponding sEVs was a cross-species phenomenon.

We further proved that upregulation of miR-27a-3p promoted proliferation and migration *in vitro*, thus contributing to the malignancy of liver cancer cells. The tumor-promoting effect of miR-27a-3p was confirmed in the nude mouse xenograft model. Over-expressing of miR-27a-3p promoted tumor cell proliferation and increased tumor formation. The most exciting finding was that knocking down miR-27 completely suppressed tumor formation. In the xenograft tumor, we also observed an increased infiltration of CD68 macrophages and CD206 immune suppressive macrophages in the miR-27a-3p over-expressing group. These findings indicated that miR-27a-3p might affect the phenotype of TAMs and inspired us to explore the effects of miR-27a-3p on the phenotype of macrophages. It turned out that the over-expressing of miR-27a-3p induced M2 polarization, while the down-regulation of miR-27a-3p reduced the expression of the M2 marker in macrophages. These observations suggest that miR-27a-3p may promote tumor progression through direct action on tumor cells and indirect regulation of tumor-related macrophages.

The increased intracellular miR-27a-3p from activated HSCs can be passed to neighboring cells via sEVs. To test whether HSC-sEV loaded miR-27a-3p can directly promote tumor progression or indirectly by educating macrophages, we adopted the concept of engineered sEVs to obtain LX2-derived sEVs that were over-expressing or under-expressing miR-27a-3p from mimic-miR-27a-3p or anti-miR-27a-3p transfected LX2 cells. Conditioned culture of HepG2 cells with miR-27a-3p over-expressing LX2-sEVs profoundly promoted the malignant behavior of tumor cells, and miR-27a-3p over-expressing LX2-sEVs pretreated macrophage showed an even more substantial effect.

Engineered sEV is a sort of sEV modified with surface decoration and/or internal therapeutic molecules. After appropriate modification, engineered sEVs can efficiently and precisely deliver drugs to target sites with fewer adverse effects of treatment (30). In the present study, the expression of miR-27a-3p in LX2-sEVs was regulated by transfection of chemically modified small nucleic acid molecules. These LX2-sEVs showed powerful effects on tumor cells and macrophages. Studies on the effects of miRNA on the malignant biological properties of tumor cells or macrophage polarization are accumulating. For example, the expression of miR-148a-3p was found to be suppressed in activated HSC-derived sEVs and to contribute to the development of hepatoma by activating ITGA5/PI3K/Akt pathway (31). Some researchers also reported the effect of miRNAs on promoting macrophage M2 polarization, including miR-519a-3p in gastric cancer (32), miR-1246 in ovarian cancer (33), miR-3591-3p in glioma (34), and miR-452-5p in hepatocellular carcinoma (35). As for therapeutic translational application, these miRNAs could be delivered as components of engineered sEVs, providing a new strategy for targeting combined immunotherapy of tumors.

**Figure 7:**
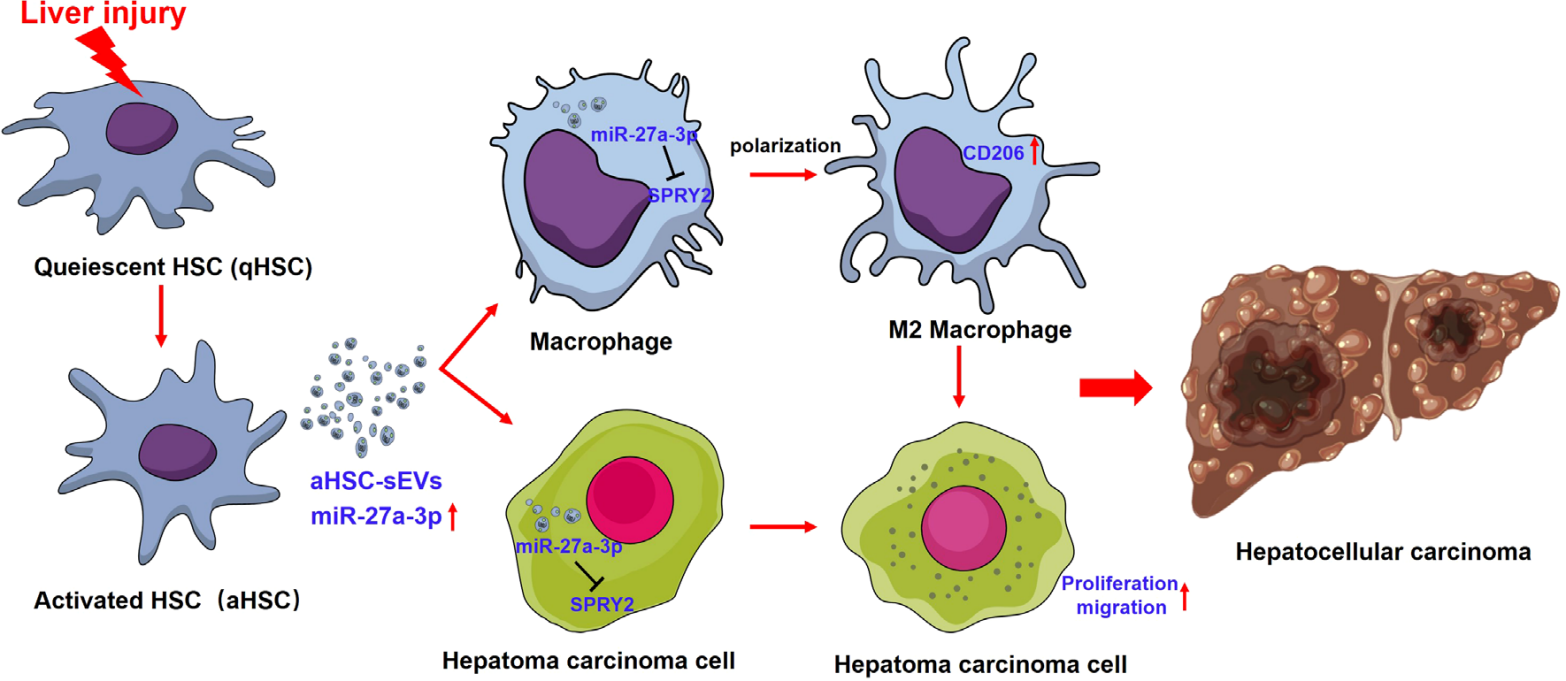
The interaction between miR-27a-3p/SPRY2 and hepatoma progression. miR-27a-3p was significantly upregulated in HSC-sEVs during the activation of HSCs. HSC-sEVs containing miR-27a-3p could promote M2 polarization of macrophage and proliferation of hepatoma cells by targeting SPRY2, leading to the progression of hepatocellular carcinoma.

In the present study, SPRY2 was identified as a target gene for miR-27a-3p. SPRY2 is an important molecule for signal regulation *in vivo*, and the deletion of SPRY2 can lead to the activation of the PI3K/Akt/mTOR and MAPK/ERK signaling pathways, promote cell proliferation and migration, and lead to the development of hepatoma *in vivo (* 36–38). Our experiments demonstrated that the increase in miR-27a-3p in both hepatoma cells and macrophages resulted in a decrease in expression of SPRY2 at the protein level. According to previous reports, the reduction in SPRY2 levels in hepatoma cells promotes the proliferation and migration of hepatoma cells, and the decrease in SPRY2 levels in macrophages leads to the polarization of macrophages toward the M2 phenotype (39). Based on the above findings, sEVs containing miR-27a-3p could promote M2 polarization of macrophage and proliferation of hepatoma cells by targeting SPRY2, leading to the progression of hepatocellular carcinoma (**Figure 7**).

## 5 Conclusions

In conclusion, miR-27a-3p over-expressing sEVs released by activated HSCs might shape an immunosuppressive tumor-promoting microenvironment by suppressing SPRY2, thereby promoting macrophage M2 polarization and the proliferation and migration of hepatoma cells, ultimately resulting in accelerated hepatoma progression. This is the first report of the functional role of activated HSC-derived sEVs with high miR-27a-3p expression in regulating macrophage function and the tumor immune microenvironment, which indicates dynamic interactions among cells during hepatoma progression. It is of particular interest that both macrophages and hepatoma cells could be targeted by activated HSC-derived sEVs and synergistically involved in the malignant progression of tumors. This study also highlights the possibility that engineered HSC-derived sEVs may be applied to treat hepatoma. However, in our subsequent study, we need to further verify the role of SPRY2, the target gene of miR-27a-3p, in the malignant biological behavior of tumor cells and the differentiation of macrophages which is now supported by the literature (36–39).

## Data Availability

The original contributions presented in the study are included in the article/**Supplementary Material**. Further inquiries can be directed to the corresponding author.
