# Supplementary material for: Activated hepatic stellate cell-derived small extracellular vesicles facilitate M2 macrophage polarization and hepatoma progression via miR-27a-3p
Source: Front Immunol. 2024 Dec 17;15:1489679. doi: 10.3389/fimmu.2024.1489679 (PMC11685157; doi:10.3389/fimmu.2024.1489679)
Supplement: Supplementary file 1 [file DataSheet1.docx]

Supplementary Material

# Supplementary Table S1

**Primers used for quantitative reverse transcription polymerase chain reaction (qRT-PCR) for mRNA and miRNA quantification**

mRNA (Homo sapiens)

| **Gene** | **Forward prime (5'-3')** | **Reverse primer (5'-3')** |
| --- | --- | --- |
| **α-SMA** | AAAAGACAGCTACGTGGGTGA | GCCATGTTCTATCGGGTACTTC |
| **GAPDH** | ACAACTTTGGTATCGTGGAAGG | GCCATCACGCCACAGTTTC |

miRNA and U6 snRNA

| **Gene** | Sense prime (5'-3') | Antisense primer (5'-3') * |
| --- | --- | --- |
| **miR-27a-3p** | TTCACAGTGGCTAAGTTCCGC |  |
| **U6** | CTCGCTTCGGCAGCACA | AACGCTTCACGAATTTGCGT |

* Sense primers for mature miRNA were provided here, antisense primer was provided by RiboBio as Universal q-PCR Primer

# Supplementary Figures


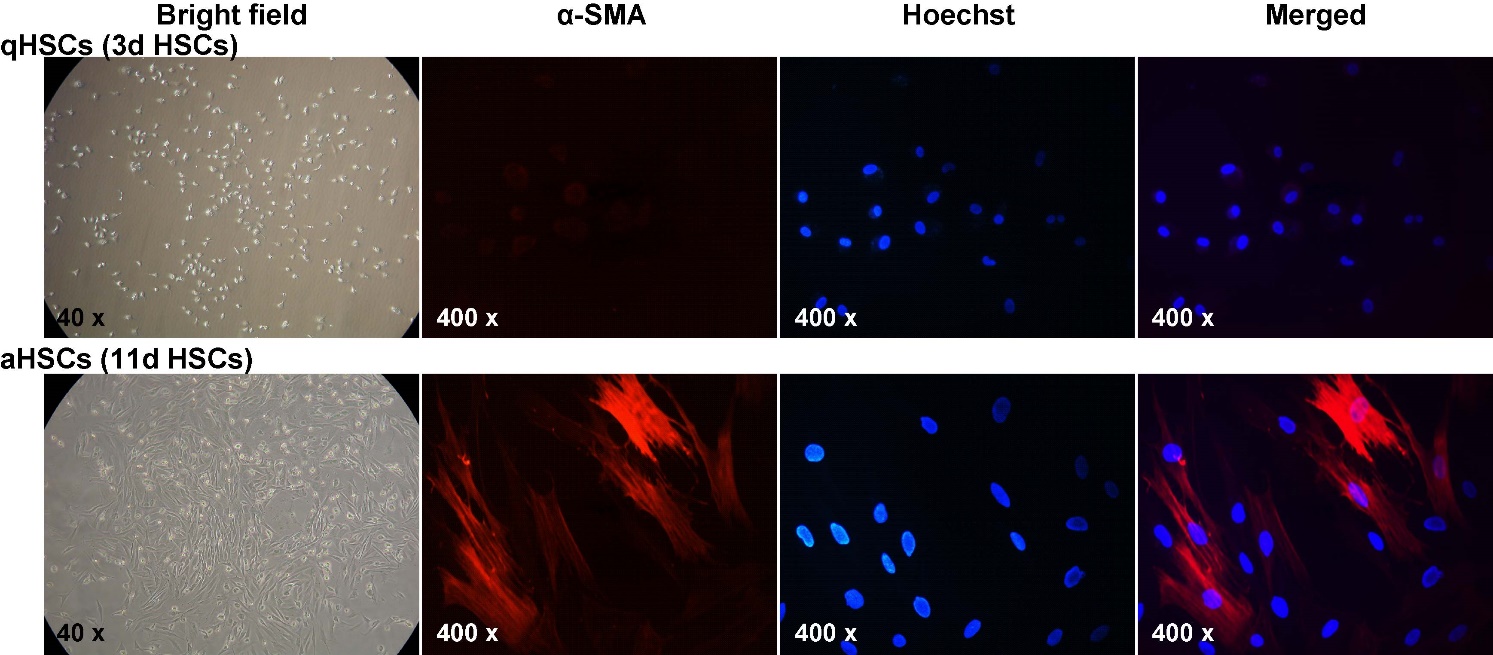


**Figure S1. Identification of primary rat hepatic stellate cells (HSCs).** Morphology of primary hepatic stellate cells of rats at day 3 (qHSCs, 3d HSCs), and day 11 (aHSCs, 11d HSCs), bright field, 40× magnification; and immunofluorescence staining of α-SMA in day 3 quiescent HSCs, and day 11 culture-activated HSCs, Cy3 (red); nuclei were counterstained with Hoechst 33258 (blue), 400× magnification. The expression of α-SMA, a key marker for HSCs activation, remarkably increased in day 11 HSCs. qHSCs, quiescent HSCs; aHSCs, activated HSCs; α-SMA, α-smooth muscle actin.

**
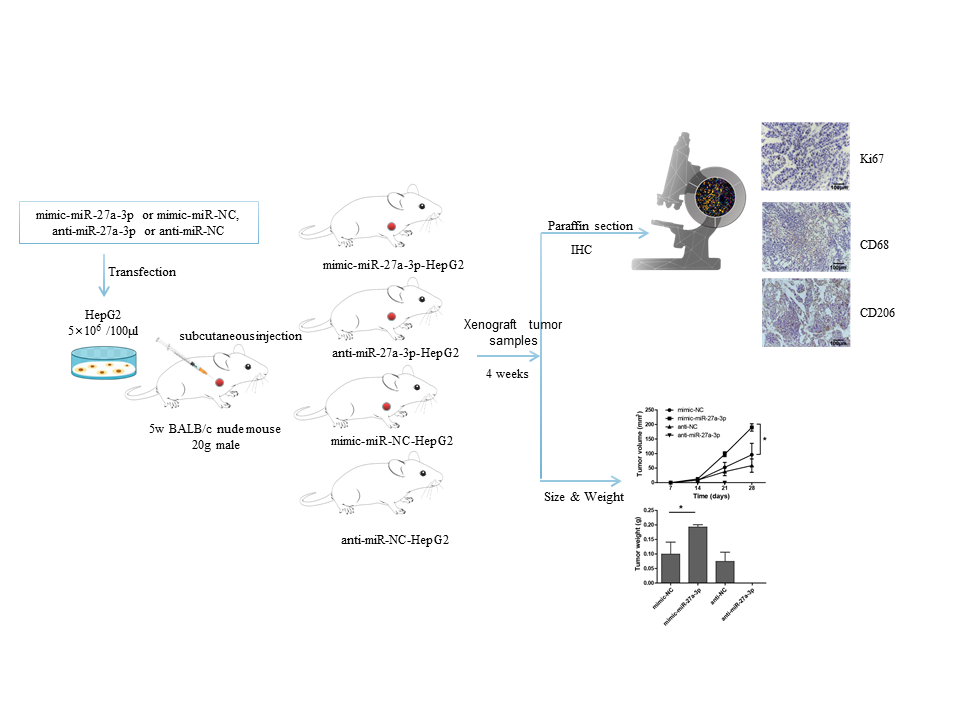
**

**Figure S2. Schematic diagram of xenograft tumor model in nude mice.** IHC, immunohistochemical staining.


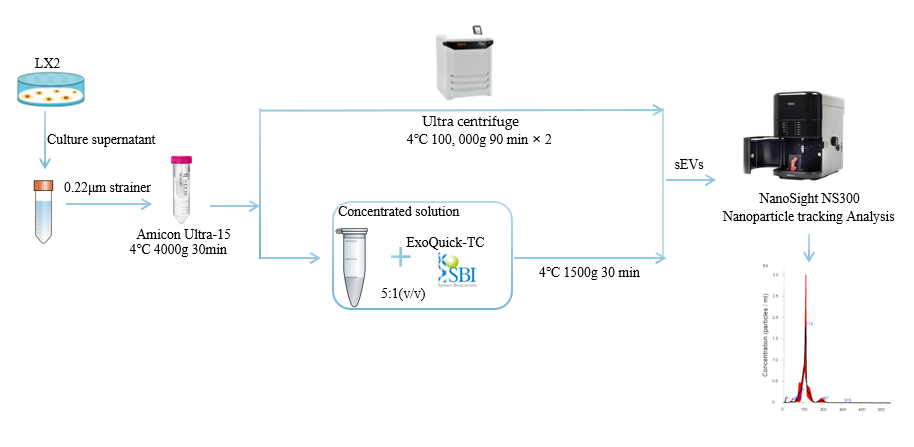


**Figure S3.** **Schematic diagram of small extracellular vesicles (sEVs) isolation in cell culture supernatant.** The overall process involves concentrating the supernatant using the Amicon Ultra-15, then separating and extracting exosomes by ultracentrifugation or using an SBI kit, and finally, measuring the number and size of sEV enriched particles using the Nanosight NS300.


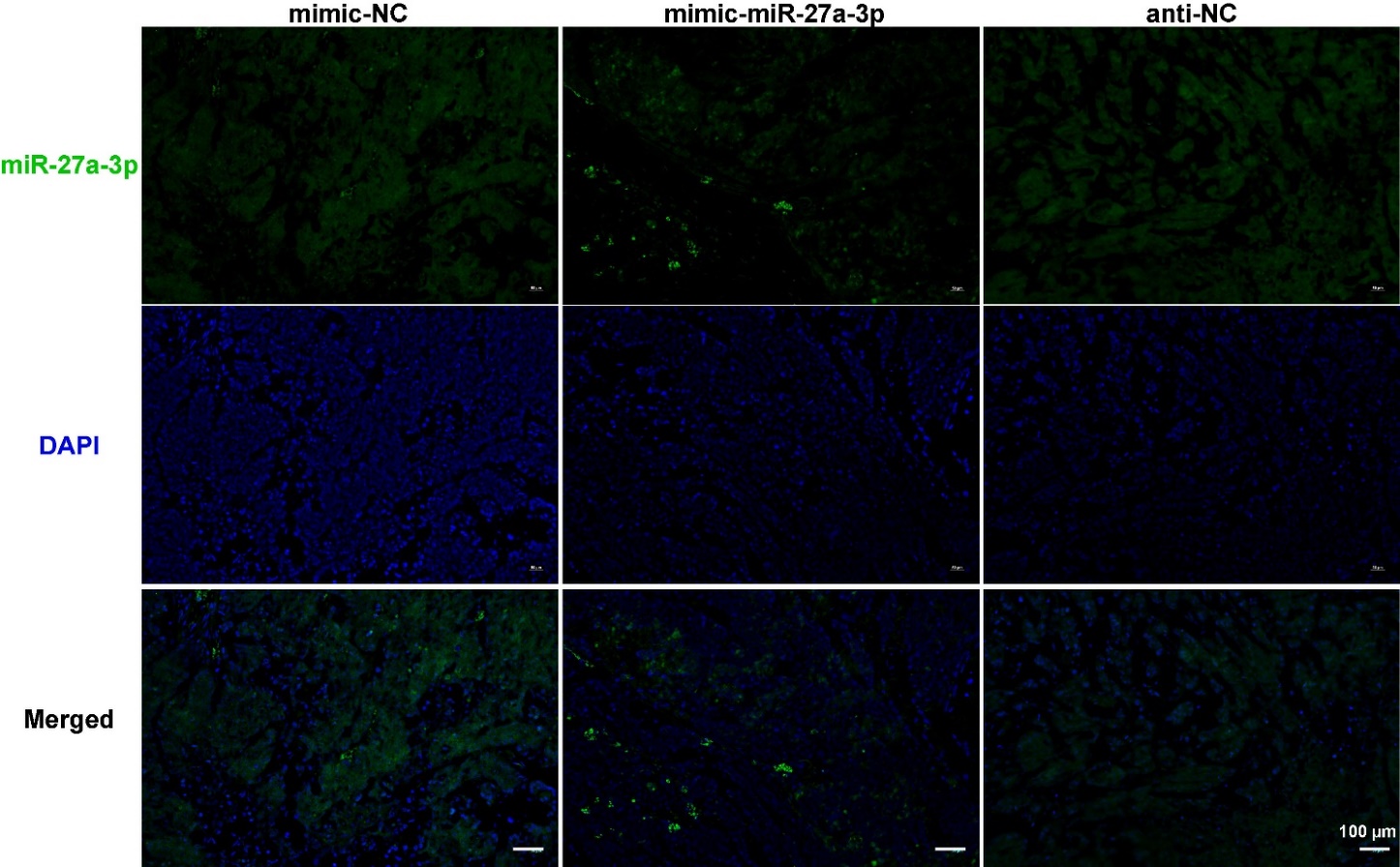


**Figure S4. Representative images for the hybridization in situ of miR-27a-3p for xenograft tumors.** miR-27a-3p, green; DAPI, blue; bar =100 μm.


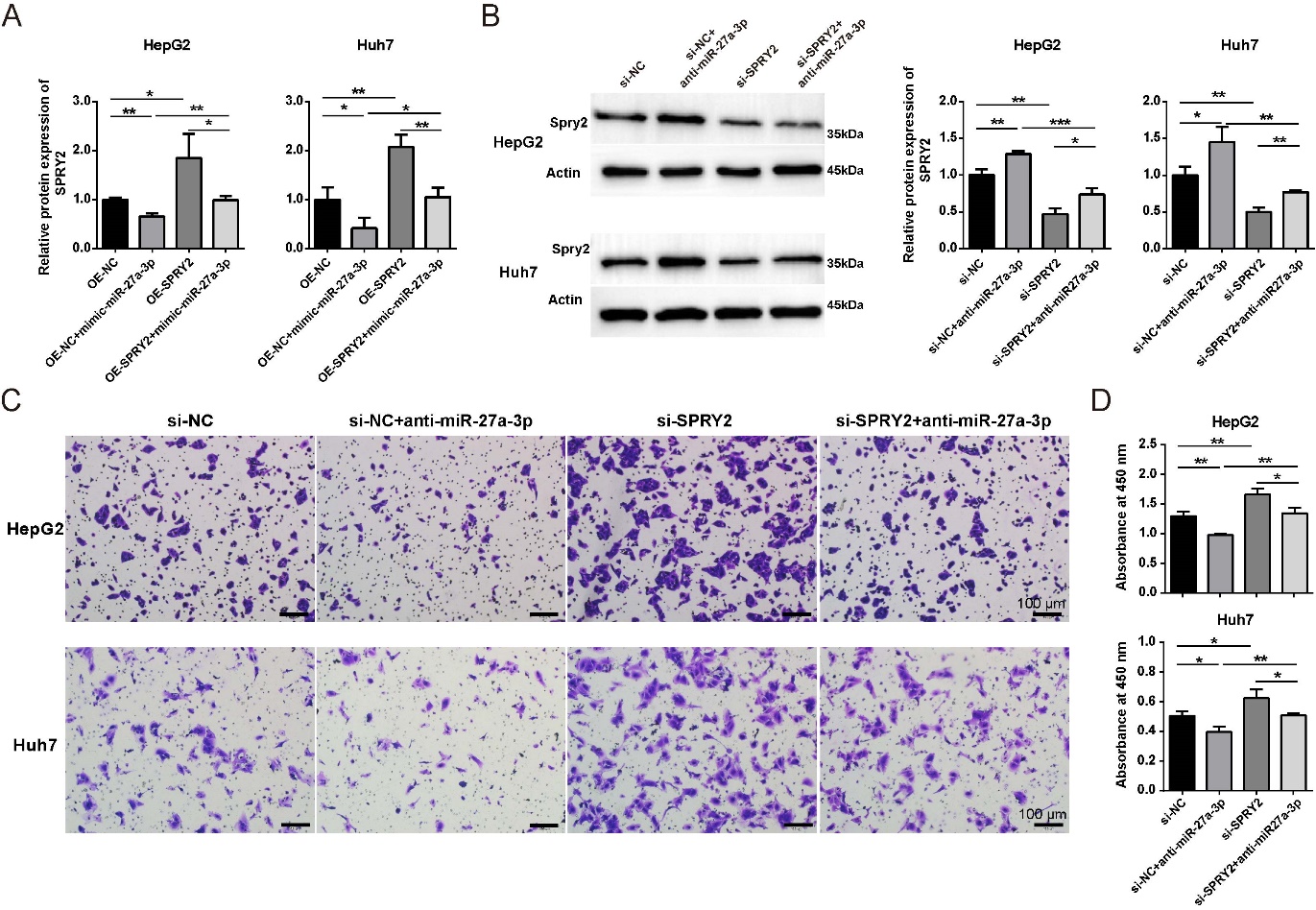


**Figure S5.** (A) The densitometrical analysis of SPRY2 protein expression by western blotting normalized to β-actin. HepG2 and Huh7 cells overexpressing SPRY2 were transfected with mimic-miR-27a-3p. (B) The expression of SPRY2 was evaluated by western blotting normalized to β-actin. HepG2 and Huh7 cells knockdown SPRY2 were transfected with anti-miR-27a-3p. (C) Transwell migration assay. SPRY2-knockdown-HepG2 and SPRY2-knockdown-Huh7 cells were transfected with miR-27a-3p inhibitor. (D) CCK8 proliferation assay. SPRY2-knockdown-HepG2 and SPRY2-knockdown-Huh7 cells were transfected with miR-27a-3p inhibitor. The absorbance at 450 nm is shown. (mean ± SEM). *P < 0.05, **P < 0.01, ***P < 0.001, ****P < 0.0001. The data were from three independent experiments.
