# Supplementary figures and images for: Activated hepatic stellate cell-derived small extracellular vesicles facilitate M2 macrophage polarization and hepatoma progression via miR-27a-3p
Source: Front Immunol. 2024 Dec 17;15:1489679. doi: 10.3389/fimmu.2024.1489679 (PMC11685157; doi:10.3389/fimmu.2024.1489679)

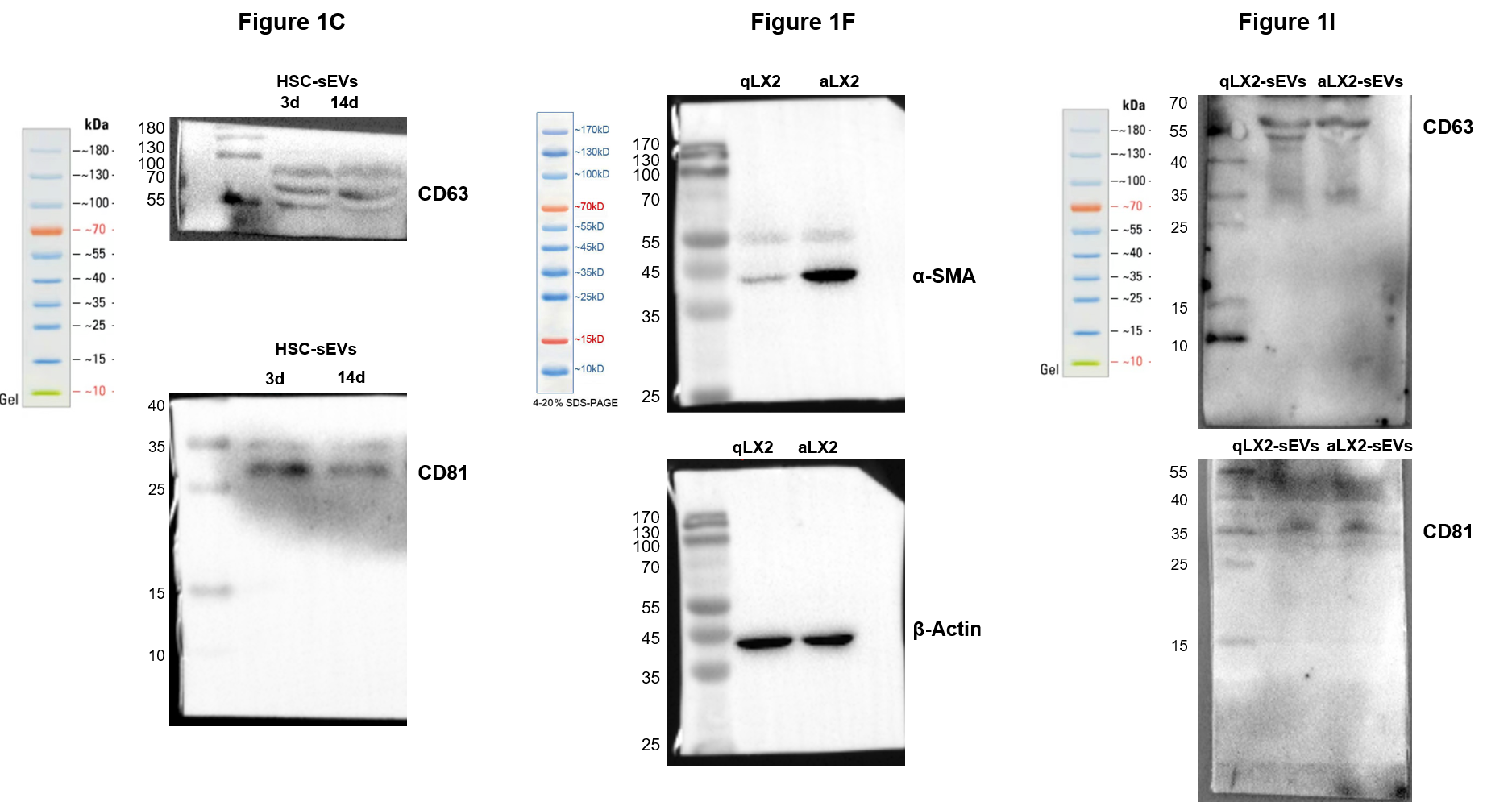

Supplement: Supplementary file 2 [file DataSheet2.zip › 新建文件夹/Figure 1.tif]

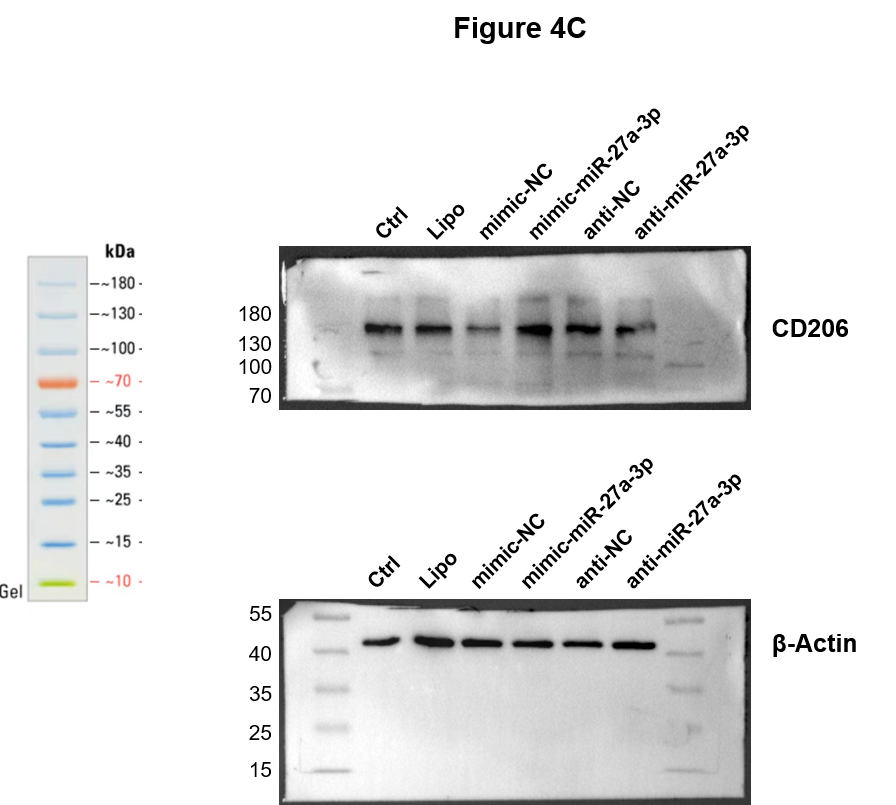

Supplement: Supplementary file 2 [file DataSheet2.zip › 新建文件夹/Figure 4.tif]

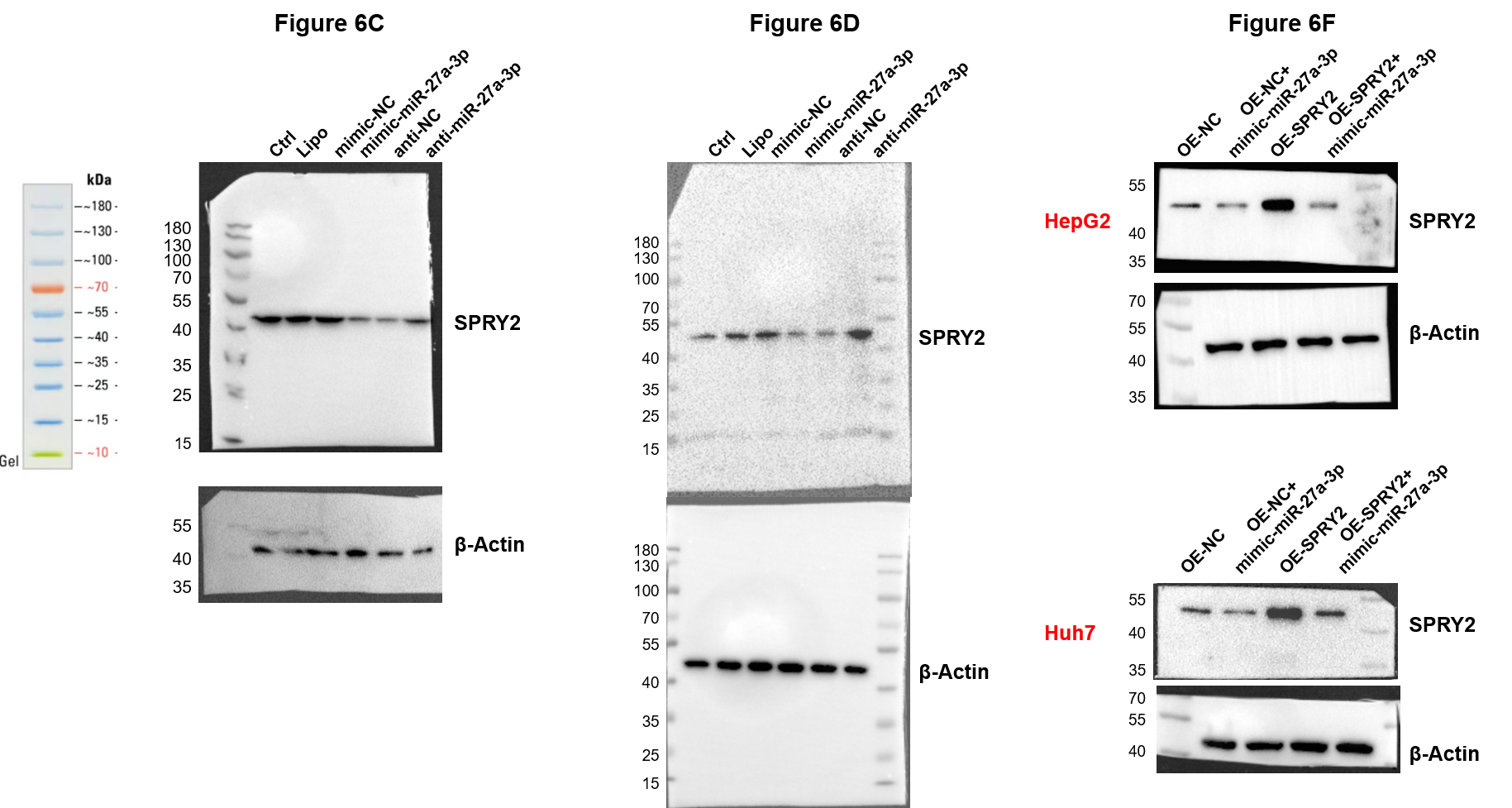

Supplement: Supplementary file 2 [file DataSheet2.zip › 新建文件夹/Figure 6.tif]

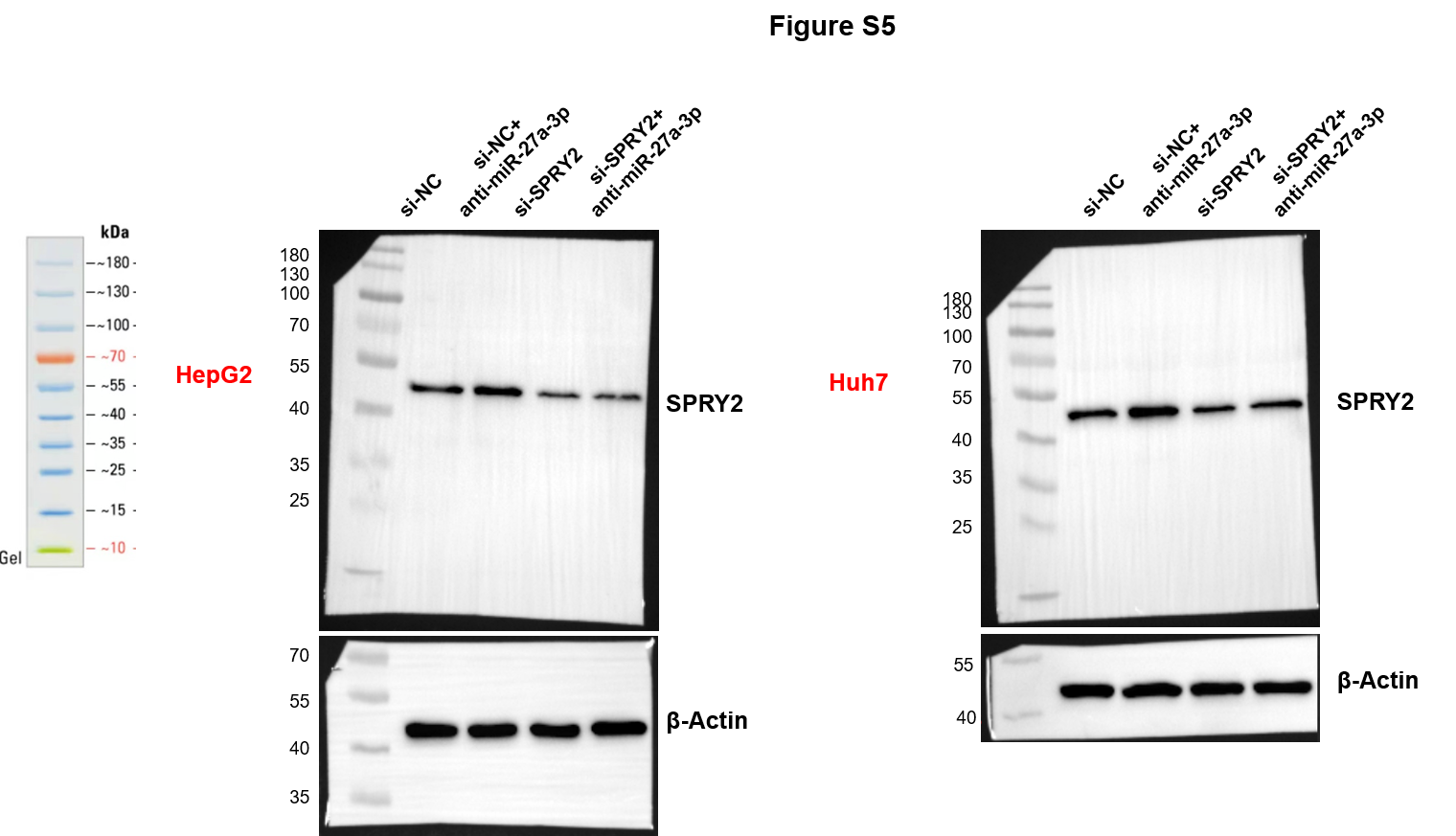

Supplement: Supplementary file 2 [file DataSheet2.zip › 新建文件夹/Figure S5.tif]
